# Diagnosis of skeletal fragility due to Loeys-Dietz syndrome and treatment with romosozumab followed by denosumab

**DOI:** 10.1016/j.bonr.2025.101849

**Published:** 2025-05-06

**Authors:** Yasutaka Tsujimoto, Naoki Yamamoto, Hayato Fukumitsu, Hironori Bando, Masaaki Yamamoto, Keiko Tanaka, Naoya Morisada, Miwako Nagasaka, Keisuke Oe, Takahiro Niikura, Mika Yamauchi, Wataru Ogawa, Hidenori Fukuoka

**Affiliations:** a Division of Diabetes and Endocrinology, Department of Internal Medicine, Graduate School of Medicine, Kobe University, Kobe, Japan; b Division of Diabetes and Endocrinology, Department of Internal Medicine, Kobe University Hospital, Kobe, Japan; c Department of Medical Genetics, Kobe University Hospital, Kobe, Japan; d Department of Pediatrics, Graduate School of Medicine, Kobe University, Kobe, Japan; e Department of Clinical and Molecular Genetics, Takatsuki General Hospital, Takatsuki, Japan; f Department of Orthopaedic Surgery, Kobe University Graduate School of Medicine, Kobe, Japan; g Department of Orthopaedic Surgery and Division of Rehabilitation, Hyogo Prefectural Nishinomiya Hospital, Nishinomiya, Japan; h Research Institute for Metabolic Bone Diseases, Eikokai Ono Hospital, Ono, Japan

**Keywords:** Osteoporosis, Skeletal fragility, Loeys–Dietz syndrome, Marfan syndrome, Romosozumab, Denosumab, Sequential therapy

## Abstract

Loeys–Dietz syndrome (LDS) is an autosomal dominant, inherited connective tissue disorder caused by a pathogenic variant in TGF-β signaling-related genes. LDS is associated with a high risk of low bone mineral density (BMD) and fractures.

We present a case report of a 43-year-old premenopausal woman with skeletal fragility who was diagnosed with LDS type 4 due to a large heterozygous deletion in the *TGFB2* gene. Upon initial referral, she was evaluated for secondary osteoporosis. Although mild abnormalities in calcium metabolism, menstrual irregularities, and lack of exercise were observed, they were not associated with this condition. However, a thorough family history and physical examination raised the suspicion of Marfan syndrome and related disorders, which were subsequently confirmed using genetic testing. Treatment with romosozumab for 1 year increased the lumbar spine BMD from 0.750 g/cm^2^ (*Z*-score −2.1) to 0.881 g/cm^2^ (*Z*-score −1.0) and the femoral neck BMD from 0.407 g/cm^2^ (Z-score − 3.0) to 0.428 g/cm^2^ (Z-score − 2.6), with a slight increase in total hip BMD from 0.525 g/cm^2^ (*Z*-score −2.6) to 0.527 g/cm^2^ (*Z*-score −2.4). Subsequent therapy with denosumab for 1 year further improved the lumbar spine BMD to 0.939 g/cm^2^ (Z-score, −0.5), femoral neck BMD to 0.496 g/cm^2^ (Z-score, −2.0), and total hip BMD to 0.552 g/cm^2^ (Z-score, −2.2). To our knowledge, this is the first case report of an improvement in BMD with romosozumab, followed by denosumab, for skeletal fragility due to LDS. Our findings suggest that this treatment regimen may be an effective therapeutic option for the management of skeletal fragility in patients with LDS.

## Highlights

• Loeys–Dietz syndrome (LDS) should be considered in the differential diagnosis of secondary osteoporosis.
• LDS diagnosis requires a detailed physical examination and family history.
• Evidence on medical treatment for skeletal fragility and osteoporosis with LDS is limited.
• This is the first report of improved BMD in patients with LDS after administration of romosozumab, followed by denosumab.

## 1 Introduction

Loeys–Dietz syndrome (LDS) is an autosomal dominant, inherited disorder caused by pathogenic variants in TGF-β signaling-related genes. LDS is a connective tissue disorder characterized by distinct cardiovascular and skeletal manifestations. Pathogenic variants of *TGFBR1*, *TGFBR2*, *SMAD3*, *TGFB2*, and *TGFB3* are reportedly causes of LDS and are classified as types 1–5, respectively (Camerota et al., 2019). The characteristic features of LDS include cardiovascular symptoms, such as aneurysms or tortuosity of the arteries, and skeletal symptoms, such as cleft palate, bifid uvula, hypertelorism, scoliosis, pectus excavatum or carinatum, arachnodactyly, and pes planus (Camerota et al., 2019). However, the subtle clinical features of LDS are often overlooked, and its rarity further contributes to underdiagnosis. Moreover, the overlapping phenotypic presentations with other Marfan syndrome (MFS)-related disorders pose challenges in achieving an accurate diagnosis.

Recently, there has been increasing awareness that LDS is associated with a high risk of low bone mineral density (BMD) and fractures. In a cohort of patients with LDS, one-third of adults had bone density *Z*-scores below −2.0. Approximately 60 % of patients with LDS experience at least one lifetime fracture (Guerrerio et al., 2022). However, evidence regarding the treatment of skeletal fragility due to LDS remains limited. Bisphosphonates are the only treatment option; however, their consistent efficacy is yet to be demonstrated (Ben Amor et al., 2012; Guerrerio et al., 2022).

Here, we present the case of a 43-year-old premenopausal woman with low BMD and a history of fracture, whose final diagnosis was LDS type 4. Sequential therapy with romosozumab followed by denosumab improved BMD. This report emphasizes the clinical course of the diagnosis and the novel treatment options for skeletal fragility due to LDS.

## 2 Case presentation

A 43-year-old woman requested bone density measurements at a local medical facility due to her parents' history of osteoporosis. She was found to have low BMD on dual-energy X-ray absorptiometry (DEXA) and was referred to our department for further investigation.

Her medical history included primary subclinical hypothyroidism and a right ankle fracture, which was considered a fragility fracture. Although she had no notable abnormalities at birth or development, she exhibited tall stature since the age of 13 years. Her menstrual history revealed menarche at the age of 15, followed by irregular cycles, although she was still menstruating at the time of presentation. She had been undergoing infertility treatment since the age of 38 and had no history of pregnancy. She had never smoked or drank alcohol but had no habit of exercising. Regarding family history, her father experienced a hip fragility fracture at a young age along with aortic regurgitation, and her mother had osteoporosis. In addition, her sister, father, and paternal grandfather exhibited a thin, tall stature. Her medication included levothyroxine 25 μg per day, as well as a cyclic estrogen and progestin regimen using chlormadinone acetate 2 mg and conjugated estrogen 0.625 mg. She also used over-the-counter natural vitamin D supplements, with no history of glucocorticoid use.

**Fig. 1:**
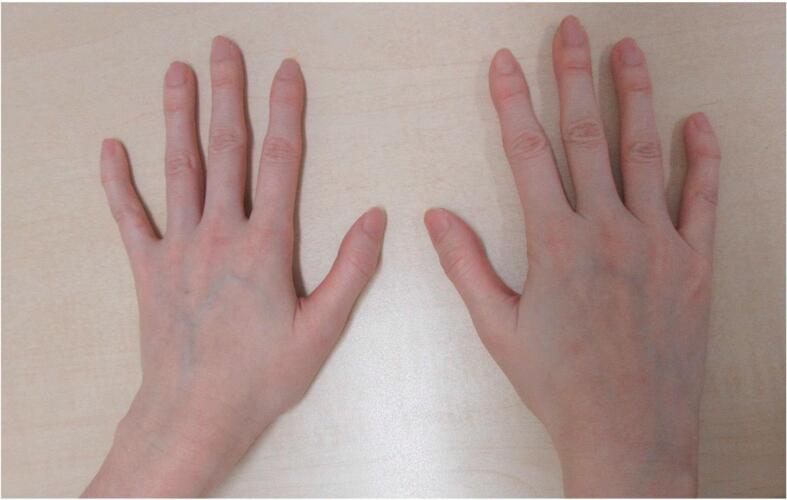
Clinical photograph of the patient's hands.

On physical examination, her height was 170 cm (+ 2.4 SD), weight was 39.5 kg, and body mass index was 13.7 kg/m^2^. Head and neck examinations revealed ocular hypertelorism and dental malocclusion. Pectus excavatum and scoliosis were found in the trunk and arachnodactyly in the extremities (Fig. 1). The results of the remaining examinations were normal. Laboratory tests revealed mild anemia with no other abnormalities (Table 1). The fractional excretion of calcium (FECa) was calculated using spot urine and corresponding serum values. No evident vertebral fractures of the thoracolumbar spine were observed on radiographs. BMD by DEXA (Discovery A, Hologic Inc., Marlborough, MA, USA) showed a lumbar spine BMD of 0.750 g/cm^2^ (*Z*-score −2.1) and a total hip BMD of 0.525 g/cm^2^ (Z-score − 2.6) (Table 2). Therefore, she was diagnosed with low bone density for age based on her history of fragility fractures and the Z-score. Despite being premenopausal, the patient's low BMD raised the suspicion of secondary causes.

**Table 1:** Laboratory findings.

		Baseline	After medical therapy	reference range
White-cell count	/μL	6600		3300–8600
Red-cell count	/μL	303⁎10^4		386-492 ⁎10^4
Hemoglobin	g/dL	9.2		11.6–14.8
Hematocrit	%	28.4		35.1–44.4
Platelet count	/μL	19.6⁎10^4		15.8-34.8 ⁎10^4
Total bilirubin	mg/dL	1		0.4–1.5
Aspartate aminotransferase	U/L	20		13–30
Alanine aminotransferase	U/L	25		7.0–23
Alkaline phosphatase	U/L	138		106–322
Albumin	g/dL	4.8		4.1–5.1
Urea nitrogen	mg/dL	10.6		280–300
Creatinine	mg/dL	0.62	0.68	0.46–0.79
Sodium	mmol/L	141		138–145
Potassium	mmol/L	3.9		3.6–4.8
Chloride	mmol/L	107		101–108
Calcium	mg/dL	10	9.6	8.8–10.1
Phosphorus	mg/dL	3.1		2.7–4.6
Magnesium	mg/dL	2.4		1.7–2.3
Glucose	mg/dL	85		73–109
Hemoglobin A1c	%	5.1		4.9–6.0
25-OH Vitamin D	ng/mL	34.1	16.5	not available⁎
intact PTH	pg/mL	72	76	15–63
FECa	%	0.3	0.33	not available
%TRP	%	85.6		not available
TSH	μIU/mL	1.6		0.61–4.23
free T4	ng/dL	1.1		0.9–1.7
anti-TPO antibody	IU/mL	<9		<16
anti-Tg antibody	IU/mL	<10		<28
anti-TSH receptor antibody	IU/L	<0.8		<2

Abbreviations: PTH, parathyroid hormone; FECa, fractional excretion of calcium; %TRP, % tubular reabsorption of phosphate; TSH, thyroid stimulating hormone; TPO, thyroid peroxidase; Tg, thyroglobulin.

⁎ 25-hydroxy vitamin D was measured using the Elecsys® Vitamin D total III assay (Roche Diagnostics). According to the manufacturer's reference ranges, deficiency is defined as <20 ng/mL, insufficiency as 20–30 ng/mL, and sufficiency as >30 ng/mL.

**Table 2:** Bone mineral density on dual-energy X-ray absorptiometry and biomarkers.

		Before romosozumab	One year after romosozumab	One year After denosumab	
L2-L4					
BMD	g/cm^2^	0.750	0.881	0.939	
*Z*-score		−2.1	−1.0	−0.5	
Femoral neck					
BMD	g/cm^2^	0.407	0.428	0.496	
Z-score		−3.0	−2.6	−2.0	
Total hip					
BMD	g/cm^2^	0.525	0.527	0.552	
Z-score		−2.6	−2.4	−2.2	
1/3 radius					
BMD	g/cm^2^	0.633	0.659	0.624	
Z-score		0.1	0.8	0.6	
TBS		not available	1.314	1.412	
TRACP-5b	mU/dL	502	200	104	(reference range: 120–420)
BAP	g/dL	10.7	13.7	6.1	(reference range: 2.9–14.5)

Abbreviations: BMD, bone mineral density; TBS, trabecular bone score; TRACP-5b, tartrate-resistant acid phosphatase 5b; BAP, bone-specific alkaline phosphatase.

The least significant change was 0.022 g/cm^2^ for the lumbar spine, 0.029 g/cm^2^ for the hip, and 0.023 g/cm^2^ for the radius.

**Fig. 2:**
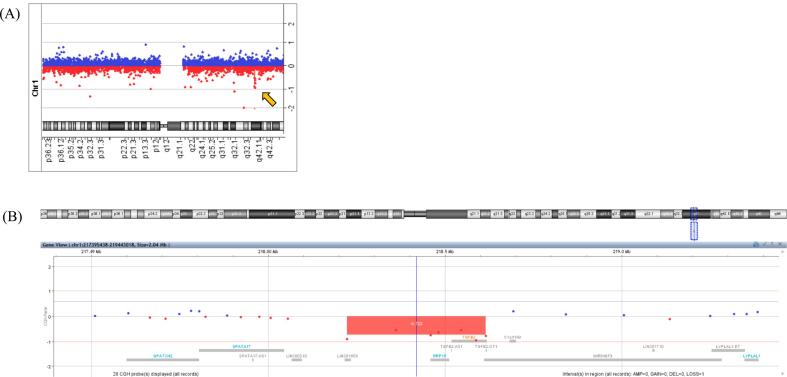
Comparative genomic hybridization (CGH) array analysis for diagnosing Loeys–Dietz syndrome (LDS) type 4 (A) Chromosome 1q profile showing a heterozygous deletion (highlighted by the arrow), identified through the CGH array. The deletion spans approximately 390 kb and includes the *TGFB2* gene, which is associated with LDS type 4. (B) Close-up view of the deleted region on chromosome 1q, illustrating the genomic location and neighboring genes. The deleted region (red box) includes *TGFB2* and overlaps with the critical region for LDS diagnosis. Data were analyzed using the CGH array software, and the coordinates were referenced based on the human genome assembly (GRCh38).

Laboratory findings showed elevated levels of tartrate-resistant acid phosphatase-5b (TRACP-5b) at 502 mU/dL (reference range: 120–420), and bone-specific alkaline phosphatase (BAP) levels were within the normal range at 10.7 g/dL (reference range: 2.9–14.5) (Table 2). Despite having a corrected serum calcium level of 10.0 mg/dL, the intact parathyroid hormone (PTH) level was mildly elevated at 72 pg/mL, raising the suspicion of normocalcemic primary hyperparathyroidism in the differential diagnosis. However, a definitive diagnosis could not be made because of the low fractional excretion of calcium at 0.3 %, even with vitamin D supplementation. Detailed physical examination revealed a thin, tall stature as well as characteristic physical and facial features. In addition, a review of the family history revealed early onset osteoporosis and valvular heart disease in her father, along with similar physical characteristics in their family lineage. Therefore, MFS was suspected to be the cause of secondary osteoporosis. Although ophthalmological examination, transthoracic echocardiography, and chest and abdominal computed tomography were performed to confirm ocular and cardiovascular complications, no abnormalities were observed. A definitive diagnosis of MFS was not reached; however, genetic testing was conducted with the patient's consent following genetic counseling because her features were consistent with MFS and related disorders. Although no pathogenic variants were identified in next-generation sequencing targeting a panel of 18 genes, including *FBN1*, *TGFBR1*, and *TGFBR2* (the full list of genes is provided in Supplementary Table 1), a heterozygous large deletion in *TGFB2* was suspected. To confirm this finding, comparative genomic hybridization array analysis was performed, which revealed a 390 kb deletion on the long arm of chromosome 1, including *TGFB2* (arr[GRCh37] 1q41(218223508_218614961)x1 (Fig. 2). On the basis of these findings, the patient was diagnosed with LDS type 4, a disorder associated with MFS.

Skeletal fragility management was discussed at a multidisciplinary conference involving endocrinologists, orthopaedic surgeons, and pharmacists. As the patient and her family expressed no plans for future pregnancies, they opted to proceed with antiosteoporotic medication to prevent further fractures. However, evidence supporting the effective treatment of MFS-related skeletal fragility remains insufficient. Although bisphosphonates are among the few treatments with established efficacy, their impact on cortical bone appears to be limited (Zebaze et al., 2014). Therefore, we decided to initiate treatment with romosozumab, aiming to promote bone formation, rapidly and reliably increase bone density, and potentially enhance the thickness of not only the cancellous but also the cortical bone. The patient and her family agreed with this approach and drug choice. Over-the-counter vitamin D supplement was switched to eldecalcitol at 0.75 μg/day, and romosozumab 210 mg/month subcutaneous injection was initiated. Exercise and nutritional therapy were provided to improve physical activity and address body weight management issues. One year after the introduction of romosozumab, biomarkers suggested an improvement in bone metabolism balance; the values for TRACP-5b decreased from 502 mU/dL to 200 mU/dL, and BAP increased from 10.7 g/dL to 13.7 g/dL (Table 2). DEXA demonstrated improvements in BMD during treatment. At the lumbar spine (L2-L4), the BMD reached 0.881 g/cm^2^ (*Z*-score −1.0) after 1 year of romosozumab therapy and further increased to 0.939 g/cm^2^ (*Z*-score −0.5) after 1 year of sequential therapy with denosumab. In the femoral neck, the BMD increased to 0.428 g/cm^2^ (*Z*-score −2.6) after romosozumab therapy and subsequently to 0.496 g/cm^2^ (Z-score − 2.0) after denosumab therapy. Similarly, in the total hip, BMD improved slightly to 0.527 g/cm^2^ (Z-score − 2.4) after 1 year of romosozumab treatment and then to 0.552 g/cm^2^ (Z-score − 2.2) following denosumab therapy. In contrast, at the 1/3 radius, the BMD remained relatively stable, increasing slightly from 0.633 g/cm^2^ (Z-score 0.1) (Discovery A, Hologic Inc., Marlborough, MA, USA) to 0.659 g/cm^2^ (Z-score 0.8) after romosozumab therapy, and measuring 0.624 g/cm^2^ (Z-score 0.6) after denosumab therapy (Horizon A, Hologic Inc., Marlborough, MA, USA) (Table 2). Additionally, the trabecular bone score (TBS), unavailable at baseline, was measured. It improved from 1.314 after 1 year of romosozumab to 1.412 after 1 year of denosumab, suggesting enhanced trabecular microarchitecture (Horizon A, Hologic Inc., Marlborough, MA, USA) (Table 2). Despite receiving nutritional counseling, the patient's body weight increased only modestly, from 39.5 kg at baseline to approximately 41 kg. No significant change in body mass index was observed. Follow-up laboratory tests were performed. The results showed a serum calcium level of 9.6 mg/dL, an intact PTH level of 76 pg/mL, FECa of 0.33 %, and a 25-hydroxy vitamin D level of 16.5 ng/mL (Table 2). Despite supplementation with eldecalcitol, the FECa remained low, further arguing against a diagnosis of normocalcemic primary hyperparathyroidism. Although conditions such as familial hypocalciuric hypercalcemia (Lee and Shoback, 2018) or acquired hypocalciuric hypercalcemia (Makita and Iiri, 2014) could account for the biochemical findings, the patient declined further diagnostic investigations. Thereafter, she continued medical therapy with romosozumab, which was re-switched after 1.5 years of denosumab treatment without any fracture events.

## 3 Discussion

Similar to MFS, LDS is associated with an increased risk of fracture (Charoenngam et al., 2023). In a study involving 77 cases of LDS, including both children and adults, low BMD, indicated by a *Z*-score of <0 on DEXA, was observed in both the lumbar spine and the total hip, and 61 % of the cases had experienced at least one fracture event (Guerrerio et al., 2022). Notably, the presence of low BMD and a history of fractures were pivotal diagnostic clues, as the characteristic physical findings had been overlooked in this case. In managing patients with LDS, it is crucial to recognize the risk of fractures and consider prevention and medical treatment.

The mechanism underlying the increased risk of fracture in patients with LDS is not yet fully understood. However, the abnormal TGF-β signaling, characteristic of LDS, reportedly plays dual roles in bone metabolism: inhibits osteoblast differentiation and promotes osteoclast differentiation. Moreover, it exerts suppressive effects on the mineralization and mechanical properties of the bone matrix (Wu et al., 2016). Although the TGF-β pathogenic variant in LDS causes a loss of function, tissue TGF-β signaling is known to be enhanced (Velchev et al., 2021). Studies on the bones of *TGF-β* knockout mice have demonstrated cortical bone thinning with limited changes in the trabecular bone (Dewan et al., 2015). Similarly, studies involving patients with LDS have revealed cortical bone thinning and a high incidence of nonvertebral fractures (Ben Amor et al., 2012; Guerrerio et al., 2022). In our case, a predominant decrease in BMD in the entire hip compared to the lumbar spine was observed. These findings suggest that an imbalance in bone remodeling in patients with LDS may lead to a decrease in BMD, particularly in the cortical bone. This mechanism appears to differ from the typical pathophysiology of postmenopausal osteoporosis. Interestingly, the baseline BMD at the 1/3 radius, a region composed primarily of cortical bone, was preserved relative to that of the lumbar spine and hip. This discrepancy implies a possible site-specific or mechanical load-dependent skeletal involvement in LDS. As the radius is a non–weight-bearing bone and the hip is a weight-bearing region with high cortical content, it is plausible that mechanical loading, in conjunction with the underlying genetic defect, may contribute to regionally distinct alterations in bone remodeling.

There is limited evidence on the treatment of skeletal fragility and osteoporosis in patients with LDS- and MFS-related diseases. It has been hypothesized that in patients with LDS, increased bone resorption leads to low BMD, and bisphosphonates have been attempted as a treatment. Although increased BMD has been observed in several reported cases (Guerrerio et al., 2022), limited effectiveness has been observed in some instances (Ben Amor et al., 2012). The reported variability in the effectiveness of bisphosphonates may be due to the unique bone metabolism in patients with LDS, which is characterized by both increased bone resorption and decreased bone formation. Therefore, sclerostin, which has a biphasic effect on bone metabolism, is a potential target for the treatment of skeletal fragility and osteoporosis in patients with LDS. Moreover, romosozumab, a monoclonal anti-sclerostin antibody, has been shown to have a dual effect on bones, increasing trabecular bone density and thickening cortical bone (Marini et al., 2022). The dual effect of romosozumab may also be anticipated in skeletal fragility and osteoporosis associated with LDS, not just in postmenopausal osteoporosis. However, the use of romosozumab is currently limited to a maximum duration of 1 year, necessitating the consideration of sequential therapy strategies. The use of denosumab leads to decreased bone resorption and increased bone density. Switching sequential therapy from romosozumab to denosumab maintains or increases bone density and reduces fractures (Cosman et al., 2016; Kendler et al., 2019). In our case, an improvement in BMD was observed after the introduction of romosozumab, and the effectiveness was maintained during sequential therapy with denosumab. Furthermore, TBS, an indirect index of trabecular microarchitecture, also improved from 1.314 to 1.412 after the sequential therapy. Although baseline TBS data were not available before treatment initiation, the post-treatment improvements suggest a favorable effect on trabecular bone quality. To our knowledge, this is the first report demonstrating the efficacy of romosozumab, followed by denosumab, in a patient with skeletal fragility due to LDS. Although the patient received nutritional counseling during the course of treatment, her body weight increased only modestly. Therefore, we consider that the observed improvement in BMD was primarily attributable to pharmacological therapy with romosozumab followed by denosumab, rather than weight gain.

LDS, an MFS-related disorder, is associated with a high risk of fractures. However, this may be overlooked in general practice. A detailed physical examination, review of family history, and genetic testing are crucial for the diagnosis of LDS. Skeletal fragility associated with LDS is characterized by a decrease in cortical bone density, and evidence of its treatment is limited. Although sequential therapy with romosozumab followed by denosumab has shown the potential to improve BMD, its effectiveness in preventing fractures remains uncertain and warrants further investigation.

The following is the supplementary data related to this article.

Supplementary Table 1 The next-generation sequencing targeting a panel of 18 genes. Supplementary Table 1

## CRediT authorship contribution statement

**Yasutaka Tsujimoto:** Writing – original draft, Visualization, Data curation, Conceptualization. **Naoki Yamamoto:** Writing – review & editing. **Hayato Fukumitsu:** Writing – review & editing. **Hironori Bando:** Writing – review & editing. **Masaaki Yamamoto:** Writing – review & editing. **Keiko Tanaka:** Writing – review & editing, Investigation. **Naoya Morisada:** Writing – review & editing, Investigation. **Miwako Nagasaka:** Writing – review & editing, Investigation. **Keisuke Oe:** Writing – review & editing. **Takahiro Niikura:** Writing – review & editing. **Mika Yamauchi:** Writing – review & editing. **Wataru Ogawa:** Writing – review & editing. **Hidenori Fukuoka:** Writing – review & editing, Supervision, Conceptualization.

## Informed consent

The patient provided informed consent for publication.

## Funding sources

This study did not receive any specific grants from funding agencies in the public, commercial, or non-profit sectors.

## Declaration of competing interest

None.

## Data Availability

No data was used for the research described in the article.
